# Unbiased anchors for reliable genome-wide synteny detection

**DOI:** 10.1186/s13015-025-00275-9

**Published:** 2025-04-05

**Authors:** Karl K. Käther, Andreas Remmel, Steffen Lemke, Peter F. Stadler

**Affiliations:** 1 Bioinformatics Group, Department of Computer Science, and Interdisciplinary Center for Bioinformatics, Leipzig University, Härtelstrasse 16-18, D-04017 Leipzig, Germany; 2 Zoology Department, University of Hohenheim, 10587 Stuttgart, Germany; 3 Max Planck Institute for Mathematics in the Sciences, Inselstraße 22, D-04103 Leipzig, Germany; 4 Department of Theoretical Chemistry, University of Vienna, Währingerstraße 17, A-1090 Wien, Austria; 5 Facultad de Ciencias, Universidad National de Colombia, Bogotá, Colombia; 6 Center for non-coding RNA in Technology and Health, University of Copenhagen, Ridebanevej 9, DK-1870 Frederiksberg, Denmark; 7 Santa Fe Institute, 1399 Hyde Park Rd., Santa Fe, NM 87501 USA

**Keywords:** Synteny, Anchor, k-mer

## Abstract

Orthology inference lies at the foundation of comparative genomics research. The correct identification of loci which descended from a common ancestral sequence is not only complicated by sequence divergence but also duplication and other genome rearrangements. The conservation of gene order, i.e. synteny, is used in conjunction with sequence similarity as an additional factor for orthology determination. Current approaches, however, rely on genome annotations and are therefore limited. Here we present an annotation-free approach and compare it to synteny analysis with annotations. We find that our approach works better in closely related genomes whereas there is a better performance with annotations for more distantly related genomes. Overall, the presented algorithm offers a useful alternative to annotation-based methods and can outperform them in many cases.

**Supplementary Information:**

The online version contains supplementary material available at 10.1186/s13015-025-00275-9.

## Introduction

The identification of orthologous sequence elements is the fundamental task in comparative genomics. Orthology assignments form the basis of most approaches in phylogenetics and phylogenomics [1–3], underly sequence annotation workflows and serve as starting points for the a wide array of downstream analyses in computational evolutionary biology. The most common, albeit not always reliable method to establish orthology is sequence similarity, most commonly by means of pairwise best matches [4–6]. Genomes, however, rapidly evolve through sequence duplications, and hence even the genome of very closely related organisms are not entirely composed of 1-1 orthologs, as exemplified by human- and chimp-specific KRAB-ZNF genes [7]. In more distantly related species, parts of the genome will have diverged beyond the detection limit of sequence similarity searches. This is a critical issue in particular for the investigation of rapidly evolving features, including some classical structured ncRNAs [8] and most long non-coding RNAs [9]. Unidentified orthologs leads to systematic biases, such as underestimated gene ages [10] and overestimates of taxon-specific innovations [11]. In case of repeat-like gene families, which in particular also include tRNAs and clusters of histone genes, sequence similarity is insufficient to establish orthology due to various mechanisms of concerted evolution that keep paralogous gene effectively identical in sequence [12].

The most common interpretation of the notion of synteny in modern genomics defines *syntenic regions* (in two different genomes or in same genome) as genomic intervals that harbour multiple homologous genomic features in preserved order and relative orientation [13, 14]. Synteny thus is defined relative to a concept of homology, which is interpreted here in the strict sense of [15] as descent from a common ancestor. Again following [15], two genes or other genomic features are orthologs if their last common ancestor was a speciation event, and paralogs if they trace back to a duplication within an ancestral genome. Although not strictly speaking part of the definition, synteny will almost always be the consequence of preserving an ancestral gene order. Synteny thus is a very strong indication of homology at the level of genome organization. This parallels the use of sequence similarity as a means of inferring homology at the level of genes and other sequence features. As a consequence, synteny can supplement similarity information in several ways: first, limiting homology searches to small regions between syntenically conserved anchors increases sensitivity because the search space is smaller. Sequence similarities that are undetectable in genome-wide comparisons may become readily observable. Paralogous loci, in particular those that are subject to concerted evolution and are thus effectively indistinguishable by sequence similarity, can can be disambiguated due to their location between unambiguous orthologous “anchors” [16]. Moreover, synteny itself has recently been discussed as source of phylogentic information [17].

The computational problem of inferring syntenic regions takes as its input ordered lists of loci for each genome or genomic region and an assignment of homologous loci, usually quantified by a measure of (sequence) similarity such as blast bit score, see e.g. [18] for a recent review of the different approaches. Software tools commonly used in comparative genomics to map synteny at a genome-wide scale, such as DAGchainer [19] and MCScanX [20], require genome annotations as input, even though the underlying algorithms are agnostic to the biological nature of the homologous loci. In practise, these methods are thus effectively restricted to protein coding genes. The focus of many studies on distant genomes, moreover, also suggest a restriction to the best conserved sequences, i.e., to protein coding genes.

For a pair of closely related genomes *G* and *H*, synteny anchors in essence describe all the DNA that inherited without duplications or deletions from their common ancestor, and this defines the unique part of their genome-wide alignment. Genome-wide alignments therefore can be used to determine orthology in this limit. Despite major progress in the last years [21], pipelines for multiple genome alignments are very resource-intensive. It is therefore of practical interest to identify synteny anchors without the need to first produce complete alignments which necessarily involves the handling of non-unique homologies. Starting from synteny anchors in fact may be a means of reducing the computational cost of genome alignment pipelines.

Synteny can be assessed by directly comparing genomic sequences, resulting in synteny plots. Unambiguous pairwise best (blast) matches then serve as “synteny anchors” identifying positions in two genomes that are unambigously orthologous to each other. This idea was used e.g. in [16] to help disambiguate orthology of tRNA genes. Annotation-based methods drastically reduce the computational efforts. A restriction to protein-coding genes, morever, allows to perform homology assessemnts at the level of amino-acid sequences. This boosts sensitivity and thus expands the phylogenetic scope. On the otherhand, the annotation-based approach limits the resolution of the synteny maps since the number of coding genes in a genome is bounded to a few ten thousands. This restriction is not necessary, however, if more closely related genomes are considered and high resolution is desirable. This is the case in particular if small local rearrangements or the emergence of genomic innovations is in the focus.

In [22] synteny anchors were considered from a more formal point of view and it was shown that potential synteny anchors comprise “sufficiently unique” sequences in each genome. That is, in order to be a good candidate for having a unique ortholog in a related species, a sequence also needs to be sufficiently different from all other loci in its own genome. This suggest that synteny detection can be split into three stages:

1. pre-computation of anchor candidates in each genome;
2. pairwise cross-species comparisons limited to anchor candidates and identification of rearrangements between two species;
3. assessment of consistent synteny across multiple species and phylogenetic placement of rearrangement events.

An efficient pre-computation of anchor candidates requires a fast means of identifying likely regions of “sufficiently unique” DNA, as well as a fast way to checking that the candidates indeed are sufficiently different from all other genomic regions. Such an approach has potential advantages: Just like annotation data, anchor candidates can be pre-computed independently for each genome and stored as simple annotation track. In contrast to gene annotations, however, anchor candidates are not biased *a priori* towards sequences with particular biological functions. Therefore, they can be expected to yield a higher and more uniform coverage. Nevertheless we cannot expect a uniform distribution for anchor candidates; after all, it has long been known that repetitive elements exhibit a non-uniform genomic distribution [23].

In this contribution we are primarily concerned with the first step of synteny detection. We further subdivide the task into two stages: (1) identification of an initial set of anchor candidates and (2) extraction of true anchors from the candidate set. For the latter, we shall see that a simple blast comparison is sufficient to verify that a piece of genomic sequence has no other similar match in the same genome. The more difficult task is the rapid identification of moderate size regions that serve as initial candidates. This task is tackled here with the help of *k*-mer statistics. We then describe an implementation of this approach which we refer to as the AncST (**Anc**hor **S**ynteny **T**ool) and compare the use of nucleic acid anchors with the use of annotation-based (protein) anchors.

## Methods

### Theory

The theoretical foundation for the anchor-based approach was sketched in the conference proceedings contribution [22]. Here we give a somewhat more formal account. A genome *G* is represented as a string over the DNA alphabet $\{A,C,G,T\}$ with an additional “line break” character marking the ends of assembly fragments (chromosomes, scaffolds, or contigs). The set *S*(*G*) of all contiguous DNA sequences present in *G* comprises all substrings of *G* that do not contain a line break as well as their reverse complements. DNA sequences are compared by a metric distance function *d*, which in practise is derived from sequence alignments.

In order to determine whether a string $w\in S(G)$ is “sufficiently unique” in the genome *G* to serve as a synteny anchor, we compare *w* to all non-overlapping subsequences. We write $G\setminus \{w\}$ for the genome with the query *w* removed from the genome in one its location of origin, which in practice is achieved by replacing *w* by “line breaks”. This is necessary since *w* necessarily has small distances to its substrings. Note that $w\in S(G\setminus \{w\})$ whenever the genome *G* contains a second copy of *w*. By definition, if $w'\in S(G{\setminus }\{w\})$, then the reverse complement of $w'$ is also contained in $S(G\setminus \{w\})$. This suggests to quantify the “uniqueness” of *w* in *G* by its distance $d(w,w')$ to all $w'\in S(G\setminus \{w\})$. In practise, $d(w,w')$ can be computed of course by means of fast, index-based method of sequence comparison. The leads us to

#### Definition 1

A string $w\in S(G)$ is $d_0$-unique in *G* if $\displaystyle \min _{w'\in S(G\setminus \{w\})} d(w,w') > d_0$.

It follows directly from the definition that if *w* is $d_0$-unique in *G* then it is also $d_0'$-unique for all $d_0'\le d_0$.

The key idea behind synteny anchors is to identify regions within a genome *G* that can be guaranteed to be unique reciprocal matches with another genome without having to compute complete pairwise comparisons of all genomes. More formally, $w\in S(G)$ and $y\in S(H)$ are reciprocal best matches if $d(w,y)<d(w,y')$ for all $y'\in S(H)$ and $d(w,y)<d(w',y)$ for all $w'\in {S(H)}$. Here we are interested in a slightly weaker property:

#### Definition 2

Let *G* and *H* be two genomes, $w\in S(G)$ and $y\in S(H)$. Then *w* and *y* are *anchor matches* if $d(w,y)<d(w',y)$ for all $w'\in S(G\setminus \{w\})$ and $d(w,y)<d(w,y')$ for all $y'\in S({H}\setminus \{y\})$.

The idea here is that *w* and *y* define unique genomic locations up to slight shifts within the alignment of *w* and *y*. The following simple observation forms the basis our approach:

#### Lemma 1

Let $w\in S(G)$ be a $d_0^G$-unique substring in the genome *G* and $y\in S(H)$ be a $d_0^H$-unique substring in *H* and assume that $d(w,y)\le \min (d_0^G,d_0^H)/2$. Then *w* and *y* form an anchor match.

#### Proof

Set $d_1{:}{=}\min (d_0^G,d_0^H)/2$. For any $y'\in S(H)$, the definition of $d_0^G$-uniqueness and the triangle inequality yields $d_0^G< d(w,w')\le d(w,y)+d(w,y')$. Thus $d(w,y)<d_1$ implies $d_0^G-d(w,y)< d_0^G-d_1 < d(w,y')$. Analogously, we obtain $d_0^H-d_1 < d(w',y)$. Since $d_0^G-d_1 \ge d_0^G/2\ge d_1$ and $d_0^H-d_1 \ge d_0^H/2\ge d_1$ we have $d(w,y)<d(w,y')$ for all $y'\in S(H{\setminus }\{y\})$ and $d(w,y)<d(w',y)$ for all $w'\in S(G{\setminus }\{w\})$. Hence *w* and *y* form an anchor match. $\square$

Synteny anchors therefore can be computed by first identifying a set $C(G,d_0^G)$ of $d_0^G$-unique sequences in each genome *G*. Different cutoffs $d_0^G$ may be used for different genomes. In the second step, anchor matches are identified as pairs (*w*, *y*) such that $w\in C(G,d_0^G)$, $y\in C(H,d_0^H)$ and $d(w,y)<d_1 = \min (d_0^G,d_0^H)/2$ accoring to Lemma 1. Empirically, it seems sufficient to ensure that *y* closer to *w* than to any $y'\in S(H\setminus \{y\})$ and *w* is closer to *y* than to any $w'\in S(H\setminus \{w\})$ by some safety margin $\varepsilon$. This amount to choosing a more relaxed threshold $d_1=\min (d_0^G,d_0^H)(1-\varepsilon )$.

### Algorithmic considerations and implementation

There are two main practical challenges in developing an annotation-free synteny detection pipleline: (1) Candidate sets $C(G,d_0^G)$ for synteny anchors must be determined without explicitly comparing each substring *w* of a fixed length $\ell$ against the rest of the genome $S(G\setminus \{w\})$. This will be achieved by a preprocessing based on k-mer distributions. (2) The comparison of the candidate sets $C(G,d_0^G)$ and $C(H,d_0^H)$ in two genomes needs to be evaluated efficiently.

#### Anchor candidates from *k*-mer counts

Similar strings are expected to contain a large fraction of common substrings than dissimilar ones. This simple consideration gives rise several related measures of repetitiveness of a sequence such as the Lempel-Ziv complexity [24] and the match complexity [25, 26], which are based on a decomposition of *G* into maximal substrings that have at least one other match in the genome, or the the length of the shortest unique substring starting at *i* [27]. The uniqueness of a substring of length *k*, on the other hand, is easily quantified as its the total number of occurrances of the *k*-mer starting at *i* in the genome:

1 $$ h_{k} (i): = |\{ j:G[j,j + k - 1] = G[i,i + k - 1]\} |.{\text{ }} $$

Its inverse, $1/h_k(i)$, serves as position-wise *mappability score*, with a value of 1 indicating uniqueness in the genome. Uniqueness in the genome, however, does not imply that there no *k*-mers that are distinct but very similar. In fact, if $k\gg 2\log _2 n$ for genome length *n*, we expect $h_k(i)$ to be small almost everywhere (except for very recently duplicated sequences). We can expect, however, that similar sequences frequently contain the same short subsequences. The similarity of a substring *w* with the rest of its genome thus should correlate with the total *genomic* frequency of *k*-mers contained in *w*. Conversely, a sequence that is very different from all other genome regions is expected to be composed of “moderate size” *k*-mers most of which are underrepresented in the genome. Moderate size here means long enough to be “informative” i.e., rare in the genome, but short enough not to be unique with very high probability. Moreover, we are interested in anchor candidates with a length $\ell$ sufficient to have unambiguously identifyable homologs. As a *measure of rareness* for the sequence window $w=G[i,i+\ell -1]$ we use the total number

2 $$  f_{{\ell ,k}} (i): = \sum\limits_{{j = 0}}^{{\ell  - k}} {h_{k} } \left( {i + j} \right)  $$

of genomic occurrances $h_k(j)$ of the *k*-mers found in the sequence window *w*. Good anchor candidates are expected to have small values of $f_{\ell ,k}(i)$.

We used GenMap [28] to count the *k*-mer frequencies $h_k(i)$. As in the preliminary data reported in [22] we use $k=13$ as default and do not allow mismatches. Assuming a uniform nucleotide distribution, this choice of *k* amounts to about 2 expected occurrances of a random *k*-mer in the *D. mel.* genome. In general we suggest to set $k\approx \log _4(n)$. We set the interval length to $\ell =500$ and consider only starting position *i* every 250 nts. Only the windows within the 15th percentile of aggregated *k*-mer frequencies $f_{\ell ,k}(i)$ are kept and overlapping ones combined. The particular choice of the cutoff is derived from preliminary computational experiments and provides – in the authors’ opinion – a useful tradeoff between sensitivity and computational cost. In order to increase the robustness of the result, we considered potential anchor candidates with different k-mer profiles, we further pre-process the candidate anchor windows by using $k=15$, $\ell =500$, starting position *i* every 250 nts and taking again the 15th percentile as well as $k=21$ allowing for up to two mismatches.

If a new candidate window overlaps with less than 30% of an existing one, we discard it and otherwise extend the existing one. In order to test the robustness of this approach we evaluated these different values of *k* and the number of mismatches. Overall we found the method to be robust. Details on the parameter optimization can found in Additional File 1.

**Fig. 1:**
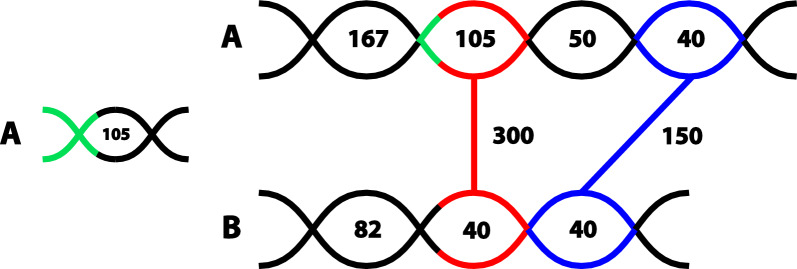
Pairwise Anchor Matches. For each anchor candidate in both genomes **A** and **B** the bit scores of the best matches in the same genome are precomputed (numbers inside the lines). Possible Anchor matches are obtained from a blast search and clasp chaining. Only matches with scores significantly better than their within-genome thresholds are retained as anchor matches

The resulting anchor candidates are then compared against the same genome *G* with blast using a word size 11 and an E-value threshold of $E=0.001$. By default, blast returns multiple matches that may be partially overlapping. In order to extract the best match of the full query sequence *w* we extracted chains, i.e., non-overlapping hits that are consecutive on both query and target [29]. The bit-scores of chained fragments are summed up to yield the total score of such as fragmented alignment. For practical computations we used the chainer with clasp [30]. The results of the chaining step were then compared with the query. All chains that substantially overlap the query are discarded and the best remaining chain, $w'$, was used to determine the level of uniqueness in the genome. In practice we divide the sequences in regions associated with a score as can be seen Fig. 1. Thus, we collect all not chained alignments which are not substantially overlapping with the original locus and assign each nucleotide in the query sequence the maximum score produced by any alignment covering it.

Since we compare an anchor candidate against possible homologs, the blast bit-score $s(w,w')$ is directly related to the corresponding metric distance measure $d(w,w')= \alpha \ell - s(w,w')$, where $\alpha$ is position-wise bit-score for self-comparison of the query of length $\ell$, i.e., $\alpha =2$ for uniformly distributed nucleotide sequences. It is possible, therefore, to use the bitscores directly without converting them to a distance measure. The end result of this preprocessing step is a set of anchor candidates *w*, each associated with similarity score $s_0(w)$ at which *w* is unique in its own genome.

The anchor candidates arguably represents a less biased set of potential synteny anchors than those employed in annotation-based approaches since we avoid the restrictions to protein coding genes, which are unevenly distribution in gene-rich and gene-poor genomic regions. This alleviated at least in part by the inclusion of non-coding elements, as shown already in [22], even though our method also recovers most protein-coding loci. We therefore also avoid the biases inherent in gene models and databases that underly automated genome annotation pipelines [31]. The analysis of the positional distribution of the anchors in Additional File 7 indeed shows that neither anchor candidates nor annotated ORFs are distributed uniformally. AncST anchors, however, consistently deviate less from a uniform distribution.

### Computations of pairwise anchors

In order to obtain anchors for a pair of genomes, we again use blast with the same parameters comparing only the anchor candidates of *G* and *H* with each other. Using $d(x,y)=\alpha \ell -s(x,y)$ yields the threshold $s(x,y)>\max (s^{G}_{0}(x),s^{0}_H)/2+\alpha \ell /2$ for a guaranteed anchor match of *x* and *y*. In practise this is overly conservative, hence we follow our preliminary work [22] and define a pair of candidates $x\in S(G)$ and $y\in S(H)$ and an an anchor match if $s(x,y)> \max (s^{G}_{0}(x)+\sqrt{s^{G}_{0}(x)}, s^{H}_{0}(y)+\sqrt{s^{H}_{0}(y)})$ as is depicted in Fig. 1. The additional square root term is introduced here to account for variance of the score estimates. If a search with *x* against the anchor candidates of genome *H* or a search with *y* as query against the anchor candidates of *G* returns more than one blast hit, we use the more conservative threshold $s(x,y)> 2\max (s^{G}_0(x),s^{H}_0(y))$. We use a minimum value of $s(x,y)=40$ in the absence significant blast hits, roughly reflecting the lower bound on significant bit scores, in order to not over-estimate the uniqueness.

Since the boundaries of anchor candidates are determined independently in each genome, there is no guarantee that anchor candidates in two genomes overlap with (nearly) there full length. This may lead to blast matches of an anchor candidate *w* in *G* with two or more non-overlapping candidates in *H* that are separated by a large genomic distance. This situation is indicative of a genomic rearrangement or an in insertion within the anchor sequence. There are two strategies to handle such cases: (i) the long anchor candidate *w* in *G* can be omitted altogether, or (ii) we may split *w* into two or more smaller anchor candidates, each matching a unique locus in *H*. In the current implementation, we omit the such candidates altogether. The outcome of this step is a set of pairwise synteny anchors that are in near perfect 1-1 correspondence.

#### Anchors for multiple genomes

Pairwise anchors computed for a set of genomes can be consolidated easily into clusters spanning multiple genomes. To this end we define the *anchor graph* whose vertices are the anchor candidates of each genome that are included in pairwise anchor. Each pairwise anchor forms an edge. Each connected component of this graph correspond to a single locus with a single ortholog in each of the genomes. Note that it is possible for a long anchor candidate in *G* to match two or more anchor candidates in *H* as long as these matches are in close genomic proximity and correspond to essentially disjoint blast alignments. In the present implementation we do not check whether such matches form a proper chain, although such as feature will likely be added in future versions of the pipeline.

In order to check the efficiency of this simple approach we check for transitivity: if (*w*, *y*) is an anchor match for *G* and *H* and (*y*, *z*) is an anchor match for *H* and *J*, then we also expect (*w*, *z*) to be an anchor match from *G* and *J*. However, the transitively implied match (*w*, *z*) is not always present. On our test set of 16 insect genomes, 30,322 of 129,155 clusters were missing some anchor matches. Overall, we found that about 1/3 of the transitively implied anchor matches were missing. This may have multiple reason. In particular, the phylogenetic distance may be large enough for a match to fall below the detection limit of blast. Another reason is the candidates differ substantially in size, with *y* being the largest, so that the overlap between *w* and *z* may be too small. To assess to what extent a lack of transitivity can be explained by such effects rather than failure of orthology, we recomputed the similarity of such implied but missing matches directly using blast and clasp and found that indeed half of the transitively inferred anchor matches do not yield alignments or only alignments with insignificant similarity scores.

**Fig. 2:**
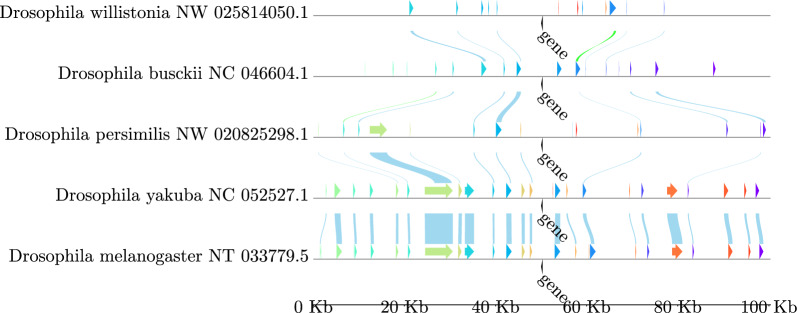
Syntenic regions of halo at a margin of 50 kb up- and downstream of all potential blastp hits of the *halo*-like genes from D. mel. Chromosome names are indicated after species names on the left and drawn as black horizontal lines with elements on them on the right. Colored elements and blue lines indicate anchors and their alignments, respectively. Alignments are drawn from pairwise alignments and anchors get the same color if they share an alignment. Genomic distances are drawn to scale. Drawn with pygenomeviz [32]

In order the test the overall reliability of the AncST pipeline, we proceeded as follows. For each inferred anchor match (*w*, *y*) with $w\in S(G)$ and $y\in S(H)$, we determined the best matches $y^*$ of *w* in the genome *H* and the best match $w^*$ of *y* in genome *G*. Again blast and clasp were used as described above to check whether our implementation correctly infers reciprocal best matches. For 14% of the anchor matches we found that *w* and $w^*$ and/or *y* and $y^*$ are separated by more than 10000nt and thus there is a better hit outside of the originally inferred syntenic region. Of these cases, about 62% were transitively inferred matches. These cases can largely be attributed to a combination of genome rearrangements/assembly artefacts and the somewhat arbitrary boundaries of our anchor candidates defined by the initial k-mer filter. This is similar to situations in which one candidate *w* in *G* finds multiple distant matches in another genomes’ set of anchor candidates. In fact, the subsequences of the anchor candidates that produce best matches outside of the inferred syntenic regions do often not substantially overlap with the subsequence originally inferred as syntenic at a median of 17% overlap of the combined length of the subsequences. In conclusion, AncST produces many high-confidence reciprocal best matches with a tolerable fraction of false positives. Future work may include the curation of the problematic cases as to define phylogenetically informed boundaries of the anchor candidates to disambiguate the matches. In practice, these cases are not to substantially influence downstream analyses because larger syntenic regions are defined by collinear chaining of clusters. Rearrangements are thus detectable as breaks in co-linearity for a subset of the genomes that are represented in the cluster. This would also make is easy to identify isolated spurious matches.

The algorithms for identifying anchor candidates, determining anchor matches, and extracting consistent anchors are implemented in AncST using python. As an example for the output produced by AncST, Fig. 2 shows the genomic region of *halo*[33]-like genes. The computation of the anchor candidates and pairwise matches for the test dataset described in Additional File 2 required less than 16 h on a HPC slurm cluster with 160 available cores. The most time intensive steps were the mappability computation with GenMap and blast searches of potential candidates against their own genomes at around 5.12 and 4.77 hours. While for this dataset the pairwise comparisons took around 2.45 hours, this step dominates rhe running time for larger datasets. The most memory intensive step is the mappability computation with GenMap. The anchors for all 16 species including information about alignments consume 646 MB of memory in total. Note that the anchor candidate computation step can be seen as preprocessing step that only need to be performed once per genome, while pairwise comparisons of annotation or anchor-candidates must be performed between all pairs of genomes.

## Comparison of annotation-based and anchor-based synteny

By design we expect AncST to work well at pairwise phylogenetic distances where large fractions of the genome are alignable as nucleic acid sequences. Clearly, no anchor matches will be detectable beyond the signficance threshold of blast alignments. At large phylogenetic distances, on the other hand, the comparison of aminoacid sequences of annotated ORFs is expected to be superior because aminoacid alignments can identify homology of highly diverged sequences. Of course, protein-based synteny detection is, by definition, limited to annotated ORFs which serve as widely-spaced anchors.

Since no ground truth is know, we compare AncST with a ORF annotation synteny assessment. We use MCScanX [20], which is probably the most widely used tool to infer collinear chains of homologs, to extract syntenic regions. MCScanX is not an end-to-end solution, but requires the homology data as input. Following the workflow described in [34] for the annotation-based approach, we performed reciprocal pairwise blast comparisons for all proteomes including self-comparisons. The respective parameters can be found in the supplement (7). The anchor matches computed by AncST are also used directly as input for MCScanX. Since the main interest is in the identification of orthologous genomic material only unambiguous alignments are considered, i.e., all pairwise collinear chains that overlap in the MCScanX output are excluded. As test data set, we use 16 genomes covering Diptera, Lepitoptera, and Coleoptera. This set includes four widely studied model species and eight additional genomes from different families with RefSeq annotations. Details on this data set can be found in Additional File 3. The computationally most expensive part in the evaluation of AncST was the blast-based comparison of annotated ORF sequences in the *MCScanX* workflow.

**Fig. 3:**
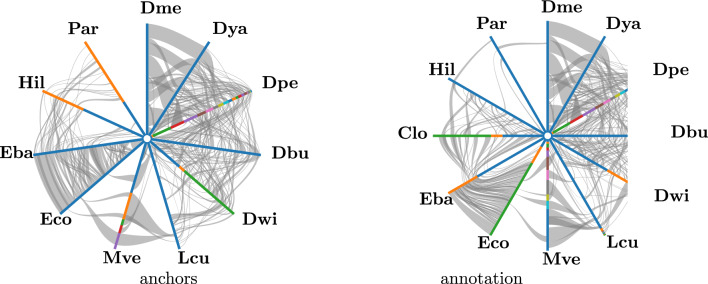
Hive view of the MCScanX collinearity file generated with synvisio [35]. L.h.s.: nucleic acid comparison with AncST (anchor-based), r.h.s.: ORF annotation based. Full species names are found in Table  2. Gray lines show pairwise alignment blocks of the connected species. Colors on genomes represent different chromosomes/scaffolds/contigs involved in the comparisons

Figure 3 give a first visual impression of the results, using Chromosome X of *D. melanogaster* as an example. Both anchor- and annotation-based data show clear regions of synteny between the five *Drosophila* species (1–5) as well as to/between *Lucilia cuprina* (6), *Musca vetustissima* (7) and *Eupeodes corollae* (8) and *Episyrphus balteatus* (9). The anchor-based approach recovers most of the annotation-based result except for the phylogenetically distant species Condylostylus longicornis and spurious matches on some contigs. Otherwise only rather minor discrepancies regarding some of the more fragmented genomes are notable.

We compare the annotation-based data and the anchor-based data using the the amount of genomic DNA in regions chained by MCScanX as performance measure. For simplicitly, we multiply the size of amino acid matches by 3 to convert them to length scales in terms of nucleotides. The total length of unambigously aligned genomic sequence is then used as performance measure for the synteny detection methods. In a addition to the fraction of the genome recovered by each method, we also assess their overlap. In order to determine the robustness of the comparisons between performance measures we considered variations of our accounting. In particular, we considered a re-evaluation of contiguous syntenic intervals produced by MCScanX, and we considered a variation of the MCScanX parameters. The details are given in Additional Files 3 and 4. Not surprisingly, the number of anchor pairs decreases with phylogenetic distance. Details are given in Additional File 5.

**Fig. 4:**
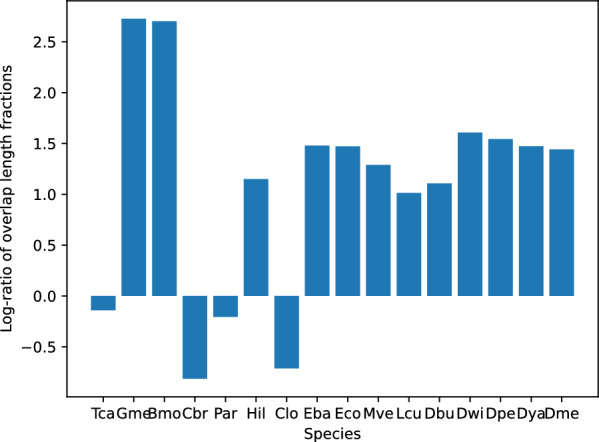
Excess of overlapping anchor/ORF lengths in filtered MCScanX co-linear chains as log-ratio of the overlap length fractions of AncST with annotated ORFs and vice versa

As expected, there are many AncST anchors in syntenic alignments which are not covered by annotated ORFs. Here we only refer to those elements of both approaches which are unambiguous aligned and thus have exactly one match in a colinearity chain in one or more target genomes. For example, AncST anchors in D. melanogaster cover about 86% of genomic positions of D. melanogaster annotated ORFs, while annotated ORFs only cover about 20% of genomic postitions of AncST anchors. Globally for the 16 species, 65% of annotated ORFs overlap with anchors at a nucleotide coverage of 69%. This matches *a priori* expectations, since a sizeable fraction of the annotated ORFs covers highly conserved regions that are too similar between paralogs to serve as anchors. Moreover, recent gene duplications, as well coding genes under concerted evolution [36] do not qualify. Conversely, 60% of all anchors overlap with annotated ORFs, at a nucleotide coverage of 29%. We observe that the unambiguous colinearity chains are more densely covered by AncST anchors ($21\pm 15\%$) than by annotated ORFs ($7\pm 9\%$). This is also reflected by differences in the length of AncST anchors (mean 1897, median 1150), compared to the length distribution of annotated ORFs in these chains (mean 907, median 626). Thus, the length measurement employed employed in Fig. 4 overestimates the overlap fraction of AncST with annotation-based ones to some extend.

In order to visualize these results per species in Fig. 4 we proceed as follows: For each species, we extract the anchors and annotated ORFs in colinear MCScanX chains and retain only elements that have unique orthologs in other species. From these subsets, we compute the relative overlap of AncST anchors with annotated ORFs and *vice versa*; thus we ask how much of the genomic positions of these anchors and ORFs are also covered by the other type. Then we take the ratio of these fractions with AncST anchors and take the logarithm as to display an excess of nucleotides of AncST anchors not covered by annotated ORFs as a positive value and vice versa as negative. In Additional File 4 a corresponding figure can be found considering not genomic positions but aligned nucleotides and the respective excesses thereof.

**Fig. 5:**
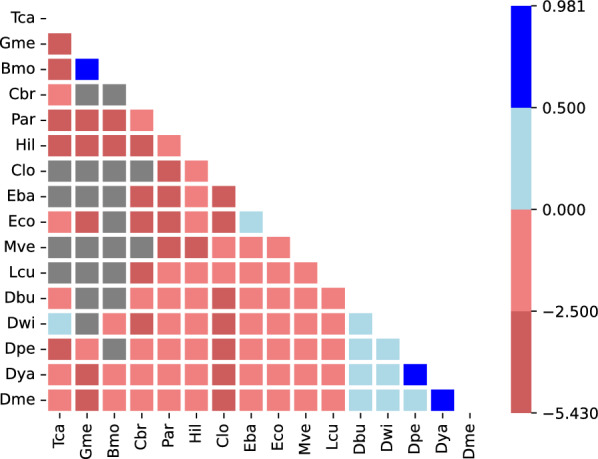
Log-ratio of the number of unambiguously aligned nucleotides between AncST- and annotation-based between two species. Blue color indicates that AncST identifies more 1-1 matches than the annotation based approach. Gray color indicates that there are no co-linear chains based on AncST

Here we opted to show the differences in aligned nucleotide lengths for all pairs in Fig. 5 since it clearly shows at which rough phylogenetic distance annotated ORFs recover more synteny blocks than AncST anchors. In summary, the annotation-based approach is clearly superior for large evolutionary distances where nucleic acid alignments become insensitive while amino acid sequences are still informative. The annotation-free anchor-based approach, which by construction operates on nucleic acid comparisons, yields better results for small and moderate phylogenetic distances where the amount of conserved non-coding DNA exceeds the genomic fraction of annotated ORFs.

The accounting used for the results outline above is deliberately designed to avoid any biases in favor of our method. Since AncST anchors are computed with the expectation that they align unambiguously, the Markov Clustering process employed by MCScanX with the given thresholds of chain length and E-value may exclude potential chains of AncST anchors, which then do not enter the chain significance calculation. Furthermore, we multiplied the length aligned protein sequences by a factor of 3 for conversion to nucleotide lengths, which is an overestimate since we ignore any mismatches in proteins. In order so see how robust these results are, we ran MCScanX with the alternative parameter settings and used an alternative, less stringent accounting strategy described in detail in Additional File 4. We observe that this skews the outcome significantly in favour of AncST anchors. We suggest that the truth about which approach covers more DNA with their alignments lies somewhere in between the overestimation of the length measurement in favour of AncST anchors and their underestimation of relative alignment length.

## Application

**Fig. 6:**
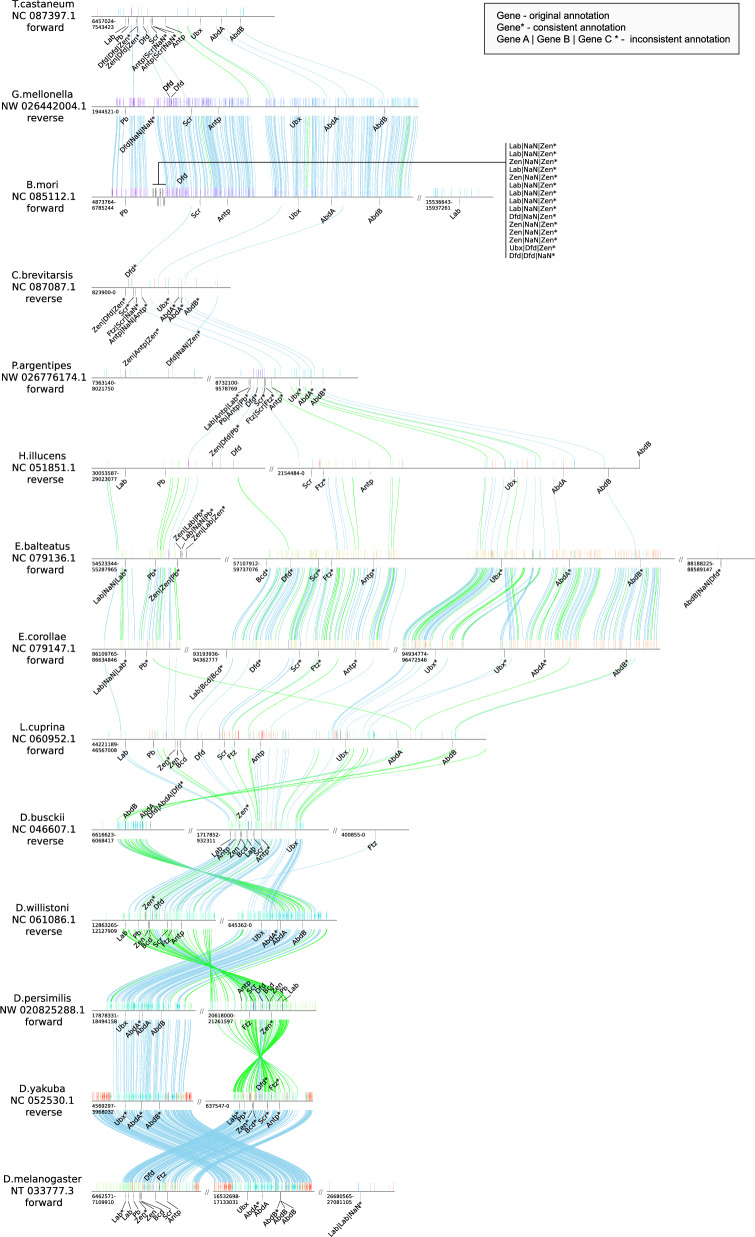
Syntenic regions of Hox gene clusters across 14 species. Annotation of loci’s gene identities (if not an original OrthoDB) was based on a combination of sequence similarity and synteny as further described and evaluated in Additional File 6. Annotations such as gene* are structured as: best blast hit | best hit according to synteny | annotation in protein tree and collapsed to one annotation if the three criteria agree. NaN indicates missing data. Genomic distances are drawn to scale. Blue lines indicate forward alignments between the anchors indicated as arrows of different colors and green lines indicate reverse alignments. The colors of the anchors are the same if they share an alignemnt. The graphic was generated with pygenomeviz [32]

As a showcase example we traced the Hox genes across the 16 species. The Hox gene cluster is well known for its highly conserved syteny in animal phyla. Nevertheless, more detailed analysis shows that exceptions are not uncommon and different lineages have variations, such as cluster breaks, inversions as well as duplications [37]. Figure 6 shows the result for species which did not display fragmentation of the resultant target regions. The full figure as well as additional tables describing the complete results can be found in the online supplement linked in section 7. The full methodology is described in Additional File 6. Expectedly, there is a dense syntenic correspondence between closely aligned species while the number of alignments drops sharply for species which are only distantly related. Nevertheless, our approach shows the usefulness of AncST anchors in (1) finding potential candidate regions and loci and (2) labelling the candidates as decribed in more detail in Additional File 6.

Many potential rearrangements can be identified directly in this figure. Some have been reported before, such as the relocation of Ubx in D. busckii [38], while others have not been notices to our knowledge: It seems like a recent shift in the order of the two Hox clusters has occurred between D. melanogaster and D. yakuba, which is likely due to inversions of the two clusters.

## Discussion and conclusions

Established methods to determine genomic synteny rely on orthologous protein coding genes and thus depend on the quality and completeness of gene annotations. As an alternative, AncST implements a novel approach that operates directly on un-annoated genomic DNA sequences. It starts from the identification of sufficiently unique DNA which can be pre-computed independently for each genome with much less effort than completing a high quality annotation of protein-coding genes. Even recent advances in homology-based annotation of coding sequences such as miniprot [39] cannot overcome that fact that only a small fraction of eukaryotic genomes is coding and annotation-based anchors thus are necessarily sparse.

The resulting anchor-candidates are then compared pairwisely between genomes using blast and a subsequent chaining of the hits. We find that this approach has the advantage of identifying significantly more syntenic DNA between closely related genomes. On the other hand, it fails for very large phylogenetic distances at which even even reading frames are no longer easy to align at nucleotide level, i.e., where the additional information of predicted protein sequences is required to establish homology. A direct comparison of the two approaches indicates that AncST performs favorably at least within the genus *Drosophila*.

The much denser map of synteny anchors computed for not-too-distant genomes provides additional power, in particular to track genomic innovations. The example of the *Hox* clusters not only traces the conservation of the gene clusters and its occasional break-ups, but also shows that the non-coding DNA between the coding regions is packed with synteny anchors.

Interesting theoretical and algorithmic questions remain for future research. Even though the *k*-mer statistics yields practically useful anchor candidates, it would be interesting to better understand its limitations and, in particular, to what extent it may overlook additional anchor candidates. Moreover, further processing of candidate anchors may be desirable: current anchor candidates may contain short subsequences that are repetitive and thus may yield spurious anchor matches. This begs the question whether anchor candidates can be locally optimized with sufficient efficiency to yield practicable improvements. We chose a statistic based on *k*-mer counts because it seems less straightforward to devise aggregate measures for moderate size intervals from Lempel-Ziv complexity and its variants, which are computed form their own intrinsic partition of the genome [26]. It will be an interesting topic for future work to see if such measures can be adapted, or even combined with *k*-mer counts, to provide a more accurate selection of anchor candidates.

The computational approach outlined here focuses on anchors that are very different from all other loci, and thus in particular excludes *near-identical* duplicated regions in the same genome. Nevertheless, genome or chromosome duplications can be detected by comparison with anchors from an non-duplicated genome if anchor candidates are compared against a target genome. Phylogenetically older duplications pose little problems as long as genomes sharing the duplication are compared: in this scenario more rapidly diverging DNA will provide unique anchors even if the most slowly evolving paralogs do not yet yield anchor sequences. Very recent duplications cannot be handled with the current approach since the duplicated sequences have not yet diverged to a point where they yield anchor sequences. It would be interesting, therefore, to extend the anchor-based approach to account for anchors with a very small number of similar matches in the same genome. A simpler, albeit not particularly elegant, approach is to subdivide a genome into smaller pieces and to check for synteny-based anchors within such regions only.

For down-stream applications it is also of interest to obtain robust multiple alignments of anchor-matches. Here we have only used the consistency of clusters of pairwise matches as a means of validating the anchor-based approach. The consistency of co-linear anchor matches could be used first to determine conserved syntenies and, in conjunction with the reconstruction of ancestral anchor-orders, also a means of tracking of local rearrangements. Regarding algorithms for such purposes, ideas such as ancestral adjacencies [40] or optimization problems considered in conjunction with clusters of phylogenetic footprints [41] might be of interest. In a similar vein, such “stacks” of orthologous anchors may also be helpful to improve multiple genome alignments since they naturally subdivide the problem in smaller alignment problems.

## Supplementary Information

Supplementary Material 1.

Supplementary Material 2.

## Data Availability

Supplementary material can be found on Zenodo (https://zenodo.org/records/13749422). The data archive contains all relevant parameter settings, the anchors and alignemnts in binary and text format as well as all necessary code and data to reproduce relevant figures as well as the underlying data in table format for the heatmaps and pygenomewiz figures. A prototype of the pipeline is available at https://github.com/Norsbus/AncST. We are also working on a website providing precomputed AncST anchors for different sets of species. A protoype is available at http://anchored.bioinf.uni-leipzig.de:8080/.
