# Supplementary material for: Unbiased anchors for reliable genome-wide synteny detection
Source: Algorithms Mol Biol. 2025 Apr 5;20:5. doi: 10.1186/s13015-025-00275-9 (PMC11972476; doi:10.1186/s13015-025-00275-9)
Supplement: Supplementary file 2 — Supplementary Material 2. [file 13015_2025_275_MOESM2_ESM.pdf]

Additional File 1

Optimization of k-mer accounting

Figure 7 displays the sum of all lengths of all pairwise alignments in an anchor set as a function of this set's k-mer accounting parameters. Table 1 gives the details on which parameters where used. First of all, bar `A` and `B` confirm the general usefullness of the `k-mer filter`. Comparing the runs differing in only the `k` used and the allowed number of mismatches it becomes clear that the method is robust to such changes. Furthermore, combining the `k-mer` counts of different `k` and `e` can improve overall performance. Further performance can be obtained by increasing the percentile of windows taken with lowest aggregated `k-mer` count, although the additional computational costs may be considered too high for some use cases.

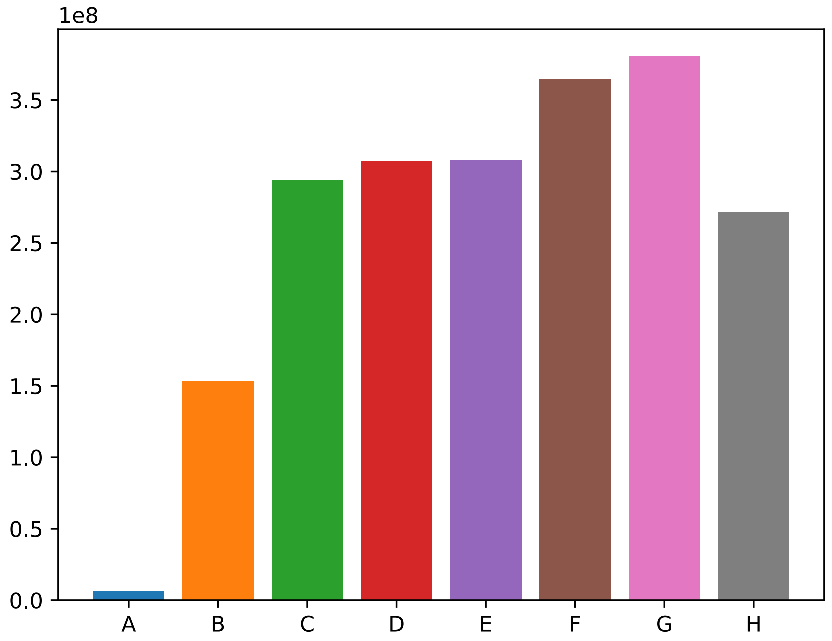

Fig. 7 Total pairwise aligned nucleotides for `AncST` runs with different parameters as described in Table 1

Table 1 Parameters used for Fig 7 as k=`k` of k-mer counting; e=number of allowed mismatches k-mer counting;p=percentile of windows taken with lowest aggregated `k-mer` count

Identifier	Parameters
A	pseudorandom sampling of windows;p=5
B	k=13;e=0;p=5
C	k=13;e=0;p=15
D	k=15;e=0;p=15
E	k=21;e=2;p=15
F	C,D,E combined as in main study dataset
G	k=13;e=0;p=29
H	C,D,E combined but with p=7

Additional File 2

Description of the test data set

The test data set comprises 16 genomes of holometabolous insects listed in Table 2.

Table 2 Genomes used sorted according to phylogenetic level.

Species	RefSeq Accession	Assembly Level	Family	model	identifier
D. melanogaster	GCF_000001215.4	chromosome	Drosophilidae	yes	Dme
D. yakuba	GCF_016746365.1	chromosome	Drosophilidae	no	Dya
D. persimilis	GCF_003286085.1	contig	Drosophilidae	no	Dpe
D. willistoni	GCF_018902025.1	chromosome	Drosophilidae	no	Dwi
D. busckii	GCF_011750605.1	chromosome	Drosophilidae	no	Dbu
Lucilia cuprina	GCF_022045245.1	chromosome	Calliphoridae	no	Lcu
Musca vetustissima	GCF_032173495.1	contig	Muscidae	no	Mve
Eupeodes corollae	GCF_945859685.1	chromosome	Syrphidae	no	Eco
Episyrphus balteatus	GCF_945859705.1	chromosome	Syrphidae	no	Eba
Condylostylus longicornis	GCF_029603195.1	contig	Dolichopodidae	no	Clo
Hermetia illucens	GCF_905115235.1	chromosome	Stratiomyidae	no	Hil
Phlebotomus argentipes	GCF_947086385.1	scaffold	Psychodidae	no	Par
Culicoides brevitarsis	GCF_036172545.1	chromosome	Ceratopogonidae	no	Cbr
Bombyx mori	GCF_030269925.1	chromosome	Bombycidae	yes	Bmo
Galleria mellonella	GCF_026898425.1	chromosome	Pyralidae	yes	Gme
Tribolium castaneum	GCF_031307605.1	chromosome	Tenebrionidae	yes	Tca

We included four model species. The remainder of the genomes was selected according the following criteria: For each of the documented families we chose the newest (on 30.04.2024) genome assembly that had a *RefSeq* annotation. If there was no species available with a respective annotation we chose the newest chromosome level assembly. *Episyrphus balteatus* was the second newest assembly as a representative of *Aschiza* because the first one was already found to be a representative of *Syrphidae*; thus it has been allocated two families in the NCBI database.

We built the phylogenetic tree in Figure 8 using the following softwares: `OrthoFinder` version 2.5.5 ([43]), `TrimAI` version v1.5.rev0 ([44]) and `IQTree` multicore version 2.1.4-beta ([45]). We used `OrthoFinder` to retrieve multiple sequence alignments of single-copy orthologues, `TrimAI` to remove all the gaps from the concatenated alignment and limit the alignment to the first 10000 amino acids and `IQTree` to compute the phylogenetic tree using the `Q.insect+R3 protein substitution model` with 1000 bootstrap iterations.

Additional File 3

Alternative accounting of `MCScanX` output

As an alternative, more comprehensive accounting, the co-linear chains extracted by `MCScanX` are directly compared by `blast` against their counterparts in the other genomes on a nucleotide level. We then remove overlapping hits from the results and take the longest possible alignments for each genome. For simplicity we do not check for collinearity in the resulting `blast` hits, which ignores local rearrangements and may slightly inflate the number of aligned nucleotides. Since the same procedure is used for both tools, this is not likely to affect their comparison, however.

Figures 9 and 10 shows that the results conform with accounting used in Figure 4 and 5, respectively. The decreased amount of excess overlap fraction of the `AncST` with annotated ORFs shows again the more densely populated `AncST`-based synteny chains compared to those based on annotations.

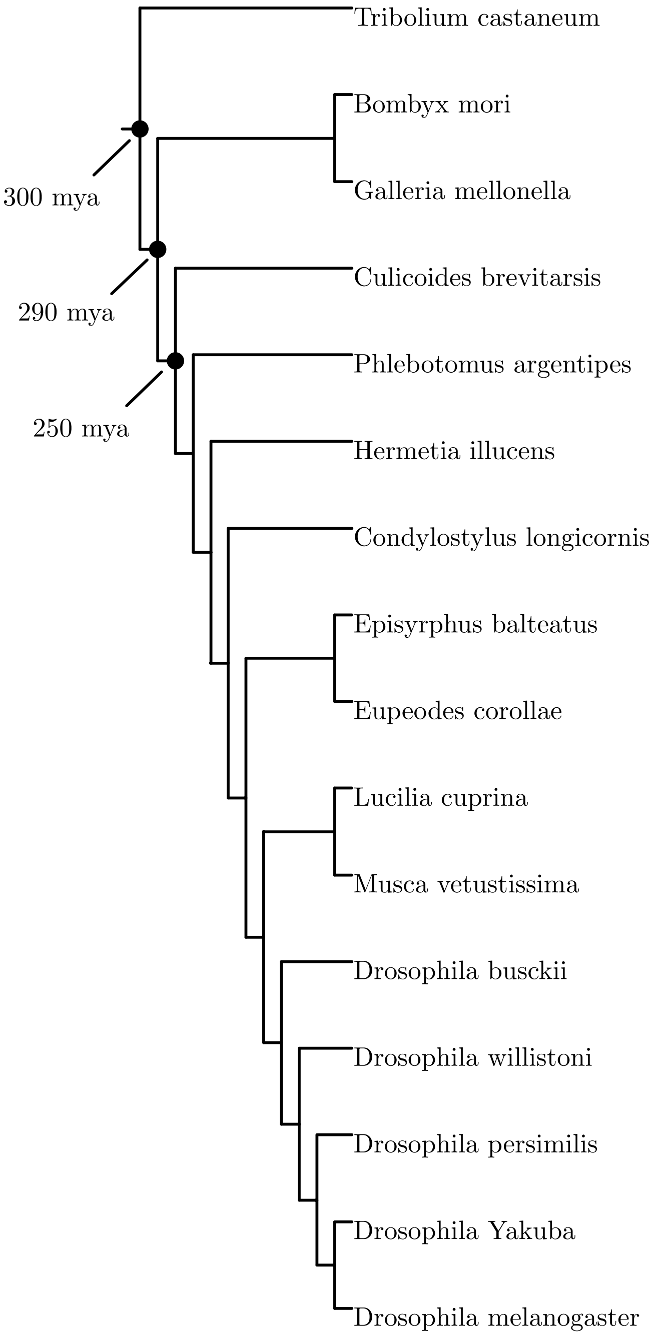

Fig. 8 Phylogeny of the 16 species based on multiple sequence alignments of single-copy genes. Estimation of node divergences from [42].

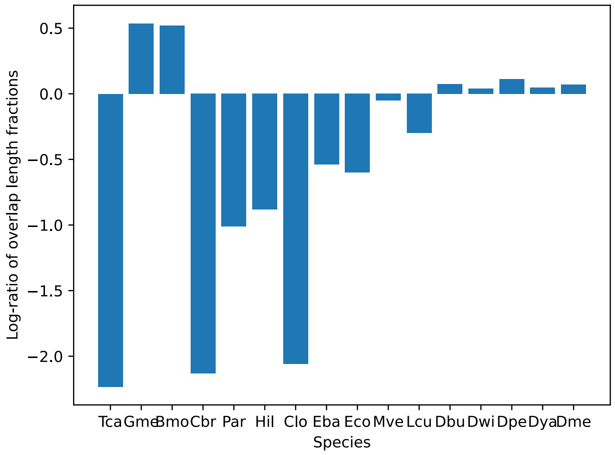

Fig. 9 Excess of overlapping alignment lengths in filtered `MCScanX` co-linear chains as log-ratio of the overlap length fractions of `AncST` with annotated ORFs and vice versa.

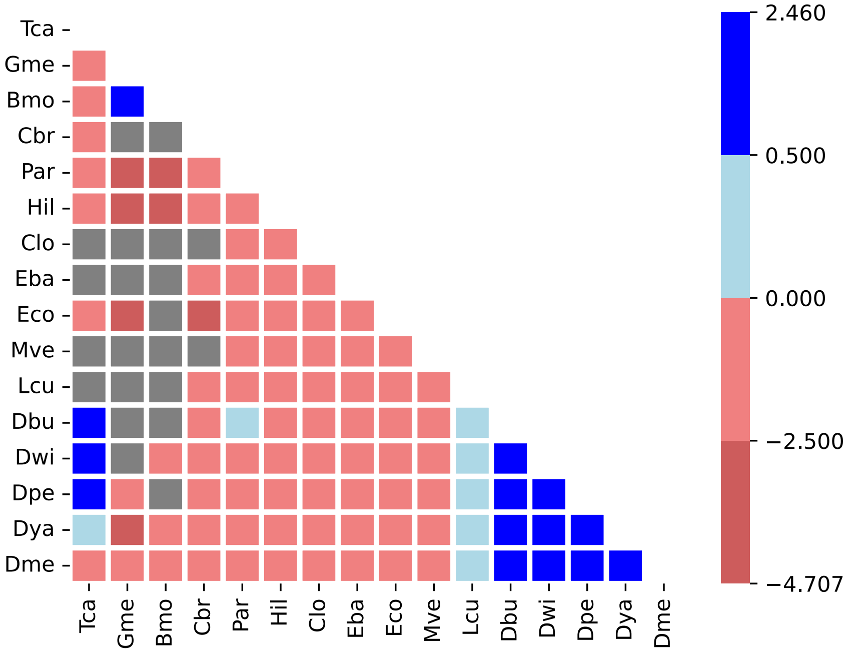

Fig. 10 Log-ratio of unambiguously aligned nucleotides between `AncST`- and annotation-based using the alternative accounting of `MCScanX` output.

Additional File 4

Adjusted MCScanX Parameters.

We ran `MCScanX` on the output of the pairwise `blast` runs with two parameter settings. The main text describes the results obtained with default parameter settings. In an attempt to optimize the performance, we modified the following parameters:

The *MATCH SIZE* parameter was decreased from 5 to 3 to include more co-linear chains. We also adjusted the E-value to 0.1 because we noticed that many of the smaller collinear chains of our anchors do not reach the default threshold of $E \leq 10^{-5}$. The E-value reported by `MSCanX` is calculated on the assumption that homologs are distributed equally on target genomes and, relying of multiplying probabilities for each matching pair, yields poor values for short chains even if the matches themselves have high confidence.

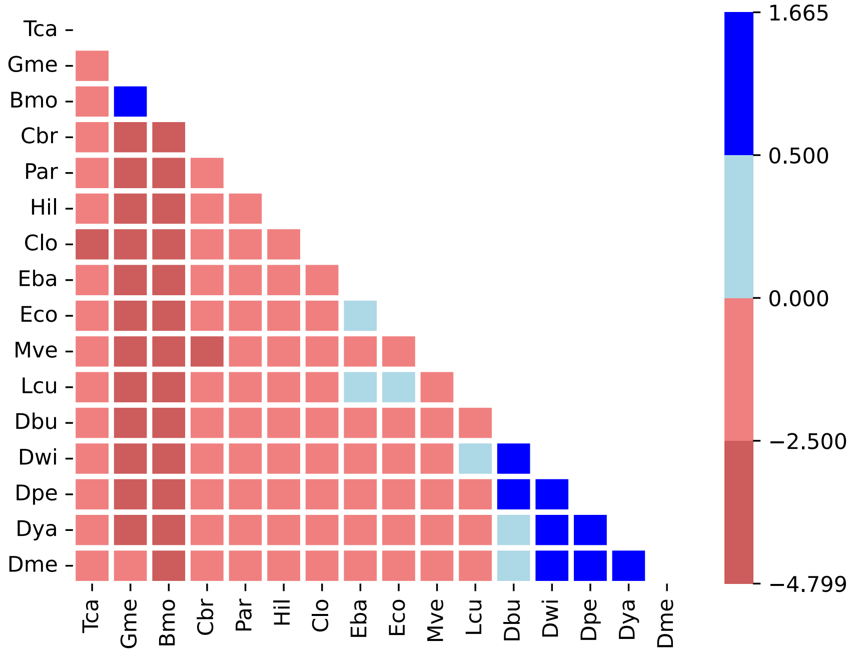

Fig. 11 Log-ratio of unambiguously aligned nucleotides between `AncST`- and annotation-based using the adjusted `MCScanX` parameters.

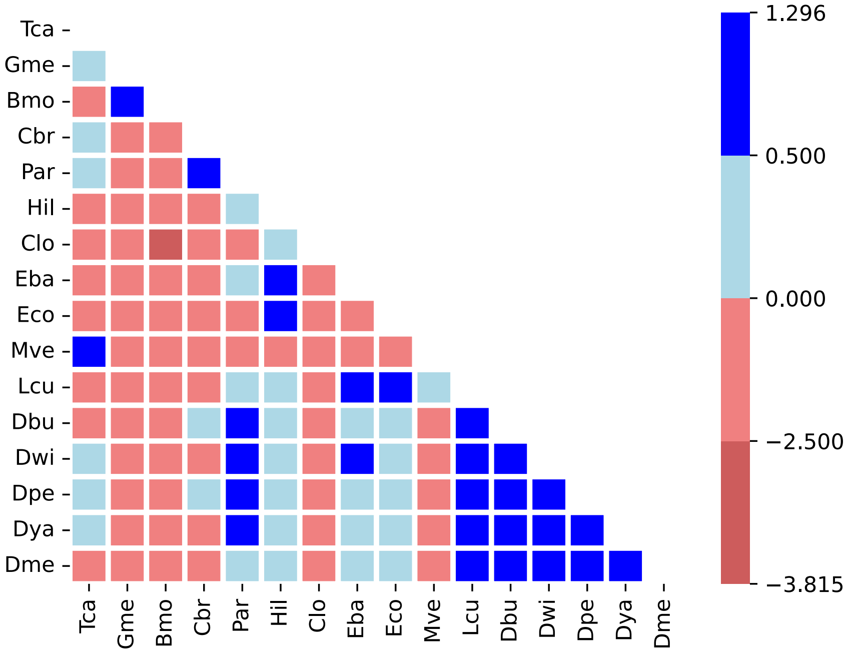

Fig. 12 Log-ratio of unambiguously aligned nucleotides between `AncST`- and annotation-based using the adjusted `MCScanX` parameters and alternative accounting of `MCScanX` output.

Figs. 11, 12, 13 and 14 show that modified `MCScanX` parameters also lead to similar results.

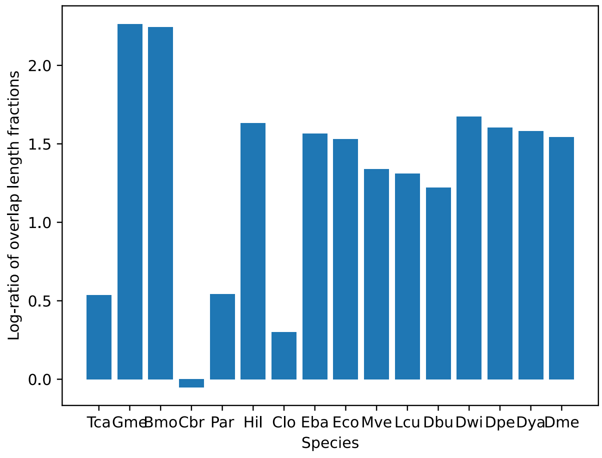

Fig. 13 Excess of overlapping anchor/ORF lenghts in filtered `MCScanX` co-linear chains from run with adjusted parameters as log-ratio of the overlap length fractions of `AncST` with annotated ORFs and vice versa.

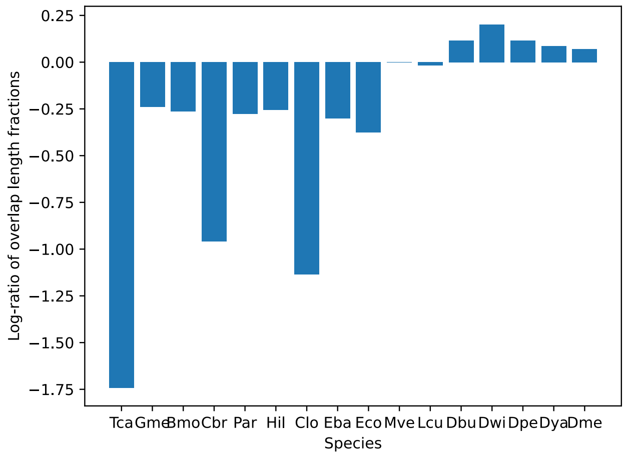

Fig. 14 Excess of overlapping alignment lengths in filtered `MCScanX` co-linear chains from run with adjusted parameters as log-ratio of the overlap length fractions of `AncST` with annotated ORFs and vice versa.

Additional File 5

Anchor pairs as a function of phylogenetic distance

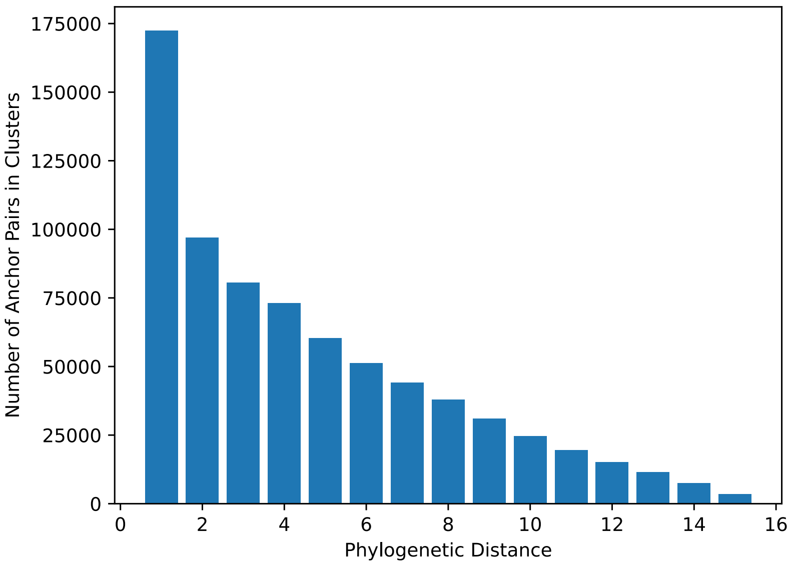

Fig. 15 Anchor pairs per `phylogenetic distance` as defnied by difference in row in Table 2.

Figure 15 depicts the number of anchor pairs in clusters when stratified by `phylogenetic distance`. For practical purposes, the phylogenetic distance simply corresponds to the separation in rows in Table 2 which is ordered phylogenetically. Although this is only a rough picture, it is clear that with growing phylogenetic distance, the amount of anchor pairs in clusters decreases. Nevertheless, there is a significant amount of anchors across a larger phylogenetic distance.

Additional File 6

We took 93 proteins annotated as `Hox genes` in `OrthoDB` and used `blasp` (`-qcov_hsp_perc` 25; `word_size` 2; `-evalue` 1e-3) to (1) find their genomic coordinates in the respective assemblies and, importantly, (2) to gather more candidate loci. Subsequently we took all `AncST` anchors 200000 bp down- and upstream of each of the `OrthoDB` loci and defined syntenic regions in the other species based on their alignments. If syntenic alignments were more than 500000 bp apart, we define two separate regions and all regions get an additional margin of 50000 bp on both sides. Then all new candidate loci are retained which are located in the respective syntenic regions. Further we labelled these candidate loci with the most likely gene annotation based on three criteria: their best blast hit among the `OrthoDB` proteins, their synteny, and

their placement in a protein tree, with `synteny` referring to the following criteria: in a first round of synteny inference only shared anchors with `OrthoDB` loci are considered while in another iteration the anchors of additional loci which were consistently identified as a particular annotation in the first round were used as well. For all loci we note the anchor closest to a locus and set as a `distance` the distance between the locus and anchor plus the distance of the closest locus to the corresponding anchor in another species to this corresponding anchor. Hence, practically we define a syntenic pair of loci if (1) there are shared anchors within the margin of 200000 bp and (2) the added distance of the respective closest anchors is minimal.
Figure 16 depicts the protein tree used in the determination of thus defined orthologs. It was calculated using combination of `MUSCLE` version 5.2.linux64 [46], `TrimAI` version v1.5.rev0 [44] and `IQTree` multicore version 2.1.4-beta [45]. We used Muscle to build the sequence alignment, `TrimAI` version v1.5.rev0 [44] to trim the alignment (custom settings: -gt 0.9 -cons 60) and to build the phylogeny with Q.insect+R3 protein substitution model and 1000 bootstrap iterations. Orthogroups were assigned manually in `Itol` [47] based on the structure of the tree and the positioning of the `D. mel` Hox genes.

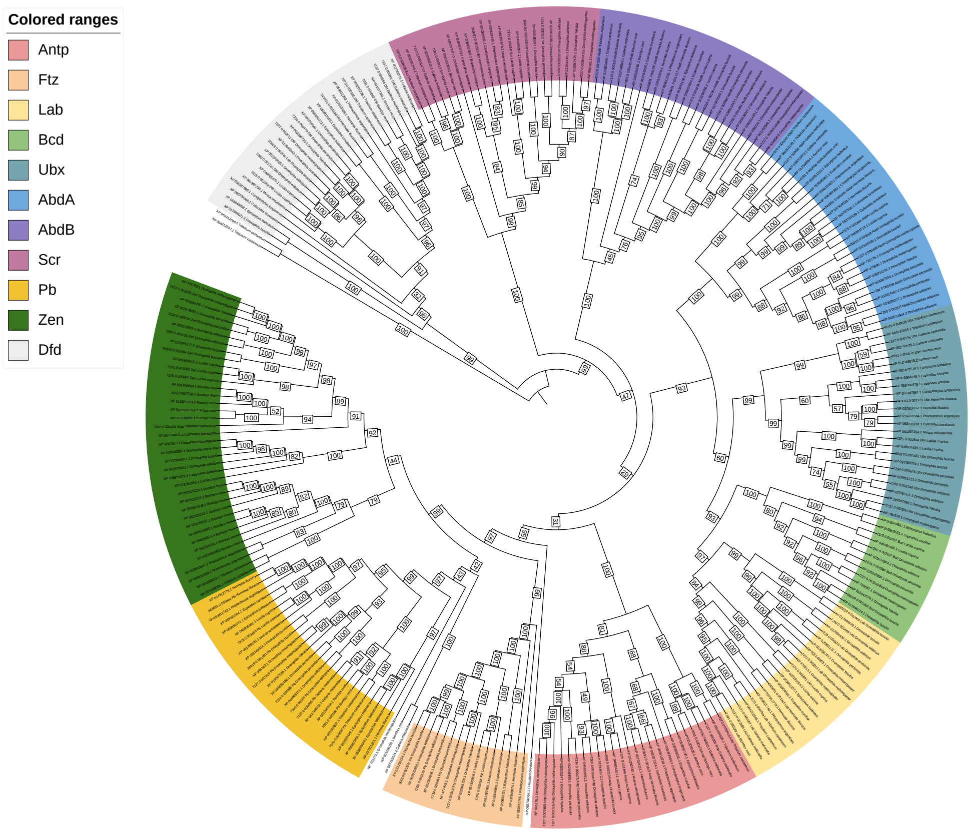

Fig. 16 Protein tree for all `Hox gene` candidates built with `MUSCLE`, `TrimAI` and `IQTree`.

Additional File 7

Table 3 Divergence measures of `AncST` anchors and annotated ORFs from uniform distribution per species for **all** anchors/ORFs. Numbers except column 1 are p-values. Input data are vectors of the midpoints of `AncST` anchors and ORFs, respectively. Midpoints are scaled to interval [0,1] where 0 represents a chromosome start and 1 a chromosome end. For KL and Chi2 data was binned into `number of data points/20` bins. KL: Kullback–Leibler divergence from uniform (KL(data||uniform)); KS_2samp: Kolmogorov–Smirnov test anchors vs. ORFs; KS: Kolmogorov–Smirnov test vs. uniform; Chi2: Chi Square test vs. uniform.

Accession	KL	KS_2samp	KS	Chi2
GCF_000001215.4	4.17e-02<br>1.26e-01	3.81e-04	2.73e-43<br>5.01e-70	3.05e-27<br>0.00e+00
GCF_016746365.1	4.59e-02<br>1.22e-01	3.15e-04	2.69e-103<br>4.27e-70	5.12e-46<br>0.00e+00
GCF_003286085.1	3.46e-02<br>8.29e-02	2.69e-02	3.18e-16<br>7.13e-18	9.47e-08<br>8.96e-244
GCF_011750605.1	3.44e-02<br>1.07e-01	3.69e-02	9.09e-07<br>9.07e-19	2.83e-07<br>0.00e+00
GCF_018902025.1	2.44e-02<br>8.71e-02	3.07e-02	5.42e-09<br>1.61e-14	9.40e-01<br>4.45e-288
GCF_030269925.1	5.63e-02<br>1.10e-01	1.33e-22	6.44e-118<br>4.72e-31	5.65e-147<br>0.00e+00
GCF_026898425.1	2.77e-02<br>7.66e-02	1.62e-03	1.06e-17<br>6.28e-08	8.11e-03<br>7.00e-272
GCF_031307605.1	4.31e-02<br>1.19e-01	3.07e-04	1.96e-18<br>3.63e-54	3.68e-20<br>0.00e+00
GCF_022045245.1	6.95e-02<br>1.55e-01	4.94e-10	2.26e-65<br>8.29e-100	8.84e-197<br>0.00e+00
GCF_032173495.1	5.14e-02<br>3.81e-02	8.85e-14	1.51e-43<br>1.60e-02	2.58e-75<br>2.05e-29
GCF_945859685.1	5.81e-02<br>1.55e-01	8.58e-44	1.11e-120<br>3.08e-30	5.10e-228<br>0.00e+00
GCF_945859705.1	5.89e-02<br>1.32e-01	1.05e-19	1.51e-114<br>1.96e-14	2.21e-212<br>0.00e+00
GCF_029603195.1	2.83e-02<br>9.81e-02	8.30e-06	6.88e-07<br>4.83e-10	3.87e-03<br>0.00e+00
GCF_905115235.1	4.96e-02<br>1.26e-01	2.12e-04	2.86e-13<br>7.59e-30	2.09e-62<br>0.00e+00
GCF_947086385.1	4.49e-02<br>9.28e-02	3.41e-01	1.58e-05<br>5.19e-10	4.76e-13<br>1.86e-262
GCF_036172545.1	5.57e-02<br>1.25e-01	2.00e-03	2.67e-17<br>1.37e-39	9.90e-47<br>0.00e+00

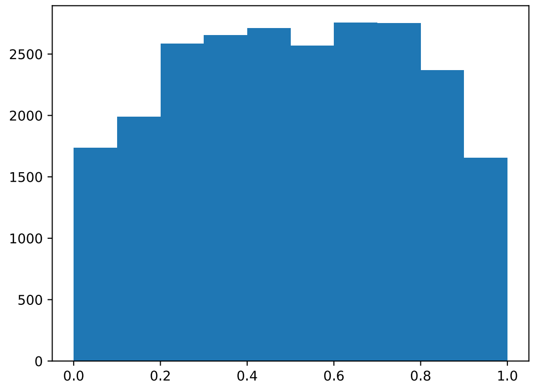

Fig. 17 Histogram of midpoints of **all** `D. mel` `AncST` anchors. Midpoints are scaled to interval [0,1] where 0 represents a chromosome start and 1 a chromosome end.

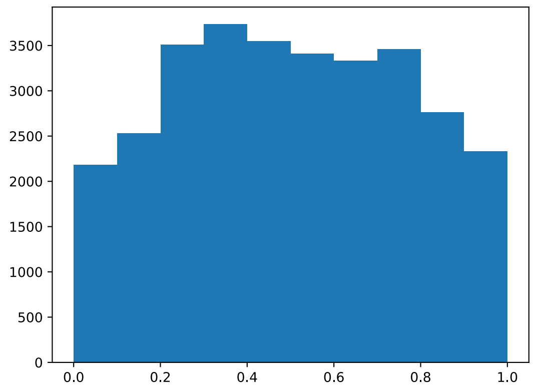

Fig. 18 Histogram of midpoints of **all** `D. mel` annotated ORFs. Midpoints are scaled to interval [0,1] where 0 represents a chromosome start and 1 a chromosome end.

Table 4 Divergence measures of `AncST` anchors and annotated ORFs from uniform distribution per species for all anchors/ORFs which are part of a non-overlapping `MCScanX` chain. Numbers except column 1 are p-values. Input data are vectors of the midpoints of `AncST` anchors and ORFs, respectively. Midpoints are scaled to interval [0,1] where 0 represents a chromosome start and 1 a chromosome end. For KL and Chi2 data was binned into `number of data points/20` bins. KL: Kullback–Leibler divergence from uniform (KL(data||uniform)); KS_2samp: Kolmogorov–Smirnov test anchors vs. ORFs; KS: Kolmogorov–Smirnov test vs. uniform; Chi2: Chi Square test vs. uniform.

Accession	KL	KS_2samp	KS	Chi2
GCF_000001215.4	4.34e-02<br>9.98e-02	2.26e-10	1.07e-45<br>1.53e-65	2.26e-31<br>5.48e-196
GCF_016746365.1	4.66e-02<br>8.47e-02	6.42e-10	3.32e-104<br>6.45e-22	2.91e-50<br>3.81e-136
GCF_003286085.1	3.98e-02<br>7.30e-02	2.47e-01	1.13e-19<br>1.45e-09	5.49e-18<br>7.32e-82
GCF_011750605.1	4.94e-02<br>8.53e-02	2.24e-01	1.55e-08<br>3.37e-08	5.25e-25<br>8.31e-93
GCF_018902025.1	3.35e-02<br>7.79e-02	2.60e-06	8.89e-04<br>3.71e-14	4.07e-09<br>4.07e-76
GCF_030269925.1	7.48e-02<br>9.02e-02	1.23e-11	1.50e-71<br>2.40e-05	1.54e-108<br>4.97e-39
GCF_026898425.1	3.17e-02<br>4.74e-02	6.68e-02	9.63e-12<br>3.16e-02	7.77e-06<br>3.27e-09
GCF_031307605.1	9.36e-02<br>1.63e-01	7.05e-04	2.66e-07<br>4.35e-09	4.22e-04<br>1.07e-28
GCF_022045245.1	1.85e-01<br>1.48e-01	1.81e-09	2.67e-68<br>3.40e-58	0.00e+00<br>1.09e-281
GCF_032173495.1	4.28e-02<br>4.57e-02	8.24e-01	6.58e-07<br>8.80e-09	1.04e-11<br>9.55e-09
GCF_945859685.1	2.31e-01<br>1.29e-01	3.42e-36	3.63e-97<br>5.76e-14	0.00e+00<br>1.42e-191
GCF_945859705.1	1.72e-01<br>1.23e-01	2.52e-36	1.96e-81<br>8.56e-20	0.00e+00<br>2.40e-212
GCF_029603195.1	9.95e-02<br>5.88e-02	1.53e-02	6.04e-03<br>6.60e-03	1.09e-10<br>1.13e-15
GCF_905115235.1	1.18e-01<br>1.06e-01	6.98e-07	1.89e-15<br>1.91e-06	1.62e-95<br>4.73e-80
GCF_947086385.1	1.15e-01<br>1.10e-01	7.50e-04	2.05e-04<br>2.02e-06	4.42e-21<br>2.67e-40
GCF_036172545.1	2.83e-01<br>1.49e-01	1.38e-03	3.30e-07<br>4.81e-08	7.66e-22<br>5.53e-45

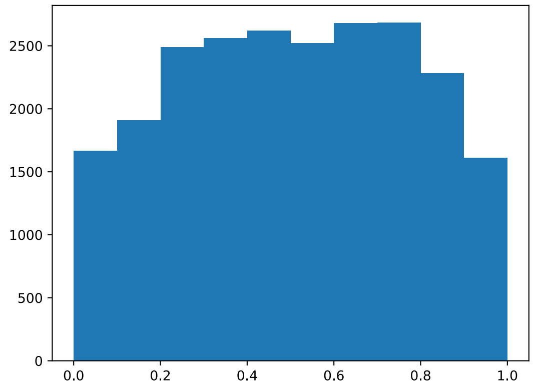

Fig. 19 Histogram of midpoints of `D. mel` `AncST` anchors which are part of a non-overlapping `MCScanX` chain. Midpoints are scaled to interval [0,1] where 0 represents a chromosome start and 1 a chromosome end.

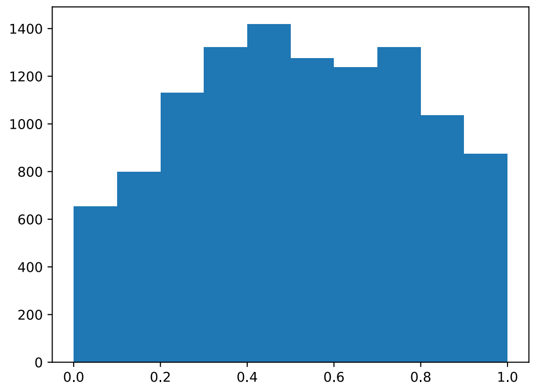

Fig. 20 Histogram of midpoints of `D. mel` annotated ORFs which are part of a non-overlapping `MCScanX` chain. Midpoints are scaled to interval [0,1] where 0 represents a chromosome start and 1 a chromosome end.
